# Endurance training facilitates myoglobin desaturation during muscle contraction in rat skeletal muscle

**DOI:** 10.1038/srep09403

**Published:** 2015-03-24

**Authors:** Hisashi Takakura, Yasuro Furuichi, Tatsuya Yamada, Thomas Jue, Minoru Ojino, Takeshi Hashimoto, Satoshi Iwase, Tatsuya Hojo, Tetsuya Izawa, Kazumi Masuda

**Affiliations:** 1 Faculty of Health and Sports Science, Doshisha University, Kyotanabe 610-0394, Japan; 2 Faculty of Human Sciences, Kanazawa University, Kanazawa 920-1192, Japan; 3 Department of Health Promotion Science, Tokyo Metropolitan University, Hachioji 192-0397, Japan; 4 Department of Biochemistry and Molecular Medicine, University of California Davis, Davis 95616-8635, USA; 5 Faculty of Sports and Health Science, Ritsumeikan University, Kusatsu 525-8577, Japan; 6 Department of Physiology, Aichi Medical University, Nagakute 480-1195, Japan

## Abstract

At onset of muscle contraction, myoglobin (Mb) immediately releases its bound O_2_ to the mitochondria. Accordingly, intracellular O_2_ tension (P_mb_O_2_) markedly declines in order to increase muscle O_2_ uptake (m
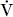
O_2_). However, whether the change in P_mb_O_2_ during muscle contraction modulates m
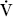
O_2_ and whether the O_2_ release rate from Mb increases in endurance-trained muscles remain unclear. The purpose of this study was, therefore, to determine the effect of endurance training on O_2_ saturation of Mb (S_mb_O_2_) and P_mb_O_2_ kinetics during muscle contraction. Male Wistar rats were subjected to a 4-week swimming training (Tr group; 6 days per week, 30 min × 4 sets per day) with a weight load of 2% body mass. After the training period, deoxygenated Mb kinetics during muscle contraction were measured using near-infrared spectroscopy under hemoglobin-free medium perfusion. In the Tr group, the m
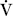
O_2_peak significantly increased by 32%. Although the P_mb_O_2_ during muscle contraction did not affect the increased m
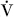
O_2_ in endurance-trained muscle, the O_2_ release rate from Mb increased because of the increased Mb concentration and faster decremental rate in S_mb_O_2_ at the maximal twitch tension. These results suggest that the Mb dynamics during muscle contraction are contributing factors to faster 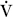
O_2_ kinetics in endurance-trained muscle.

Relative to control muscle, endurance-trained muscle increases O_2_ consumption at the same level of maximal voluntary contraction (MVC) and increases maximal O_2_ consumption, which is considered an indicator of improved aerobic exercise capacity. The increased O_2_ consumption in the trained skeletal muscle depends on both O_2_ utilization and vascular O_2_ supply. Muscle O_2_ utilization capacity is mainly determined by mitochondrial function and the quantity of mitochondria, whereas O_2_ supply capacity to the mitochondria is determined by capillarization. Many studies have reported that endurance training upregulates mitochondrial function, and mitochondria number and volume^1^,^2^,^3^. It also increases capillarization^1^,^2^,^3^. However, the contribution of O_2_ diffusion from the capillary to the mitochondria is still unknown, especially with respect to the intracellular factors involved in O_2_ transport from the sarcolemma to the mitochondria.

Recent studies have shown that the O_2_ gradient can contribute to the enhanced O_2_ flux to meet the increased muscle O_2_ demand during contraction^4^. The O_2_ saturation of Mb (S_mb_O_2_), which reflects the intracellular O_2_ tension (P_mb_O_2_), decreases as work intensity and muscle oxygen consumption (m
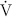
O_2_) increase. The decreasing P_mb_O_2_ expands the O_2_ gradient from the capillary to the muscle cell to increase the O_2_ flux from the vasculature to the mitochondria. Whether the O_2_ gradient contributes to the increased O_2_ uptake in endurance-trained muscle remains uncertain. With the experimental model to investigate the intracellular O_2_ dynamics^4^, we have hypothesized that the increase in O_2_ consumption in endurance-trained skeletal muscle is accompanied with increased expansion of the O_2_ gradient across the plasma membrane^4^, because studies have already shown that the change in P_mb_O_2 _during exercise can play a key role in 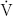
O_2_ regulation^5^,^6^.

Because training also induces an acceleration of 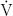
O_2_ kinetics at the onset of muscle contraction, which pulmonary 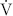
O_2_ measurements have detected and have attributed to adjustments of oxidative metabolism at the skeletal muscle level^7^,^8^,^9^, a faster 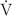
O_2_ on-kinetics could be an important adaptation, as it would potentially incur a smaller O_2_ deficit. Previous studies have already shown Mb contribution to the intracellular O_2_ dynamics, which affects the m
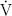
O_2_ response at the onset of muscle contraction^4^,^5^,^10^,^11^. Nuclear magnetic resonance and near-infrared spectroscopic (NIRS) experiments clearly show that Mb immediately releases O_2_ at the onset of muscle contraction and provides the initial O_2_ supply to support the rapid increase in m
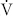
O_2_^4^,^5^,^10^,^11^. The sudden increase in m
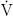
O_2_ does not appear to depend on muscle adenosine diphosphate concentration and thereby implicates a direct and immediate role for Mb-mediated O_2_ delivery^5^.

By using a hemoglobin (Hb)-free rat hind limb perfusion model, the present study shows that at all relative levels of MVC, the m
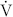
O_2_ of endurance-trained muscle exceeds that of the control muscle. Trained muscle also reaches a higher peak m
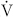
O_2_. Even though the P_mb_O_2_ of both the control and endurance-trained muscle decreases with increasing exercise intensity, the O_2_ gradient from capillary to the mitochondria does not change significantly to accommodate the differences in the observed m
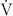
O_2_. At any given MVC, the endurance-trained muscle exhibits a smaller O_2_ gradient. However, the endurance-trained muscles exhibit a faster release of O_2_ from Mb at the initiation of contraction, consistent with the enhanced m
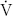
O_2_ and with the Mb-mediated O_2_ supply. Indeed the kinetics of O_2_ release from Mb can serve as an index of the change in intracellular m
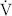
O_2_ as muscle undergoes training^5^.

## Results

Descriptive data for muscle weight are presented in Table 1. Although endurance training caused a significant reduction in body and muscle mass, the ratio of muscle mass to body mass differed slightly between the groups (with a difference in mean value of 0.1%).

Table 2 shows the contractile and metabolic properties of the control and trained hind limb muscles. Although both groups showed no significant difference in m
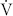
O_2_ at rest, at maximal tension, the values of the peak m
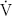
O_2_ per gram per minute in the swimming training (Tr) group were significantly higher than those in the control (Con) group. [Mb] and citrate synthase (CS) activities in the deep portion of gastrocnemius muscle significantly increased after endurance training, whereas the lactate-to-pyruvate ratio (L/P) decreased at peak maximal twitch tension. Table 3 summarizes muscle tension, the net increase in m
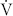
O_2_ due to muscle contraction (Δm
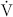
O_2_), O_2_ cost, and kinetics parameters for S_mb_O_2_ and P_mb_O_2_ at each tension level for both groups. Muscle tension, Δm
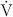
O_2_, and O_2_ cost increased as work intensity increased.

**Figure 1:**
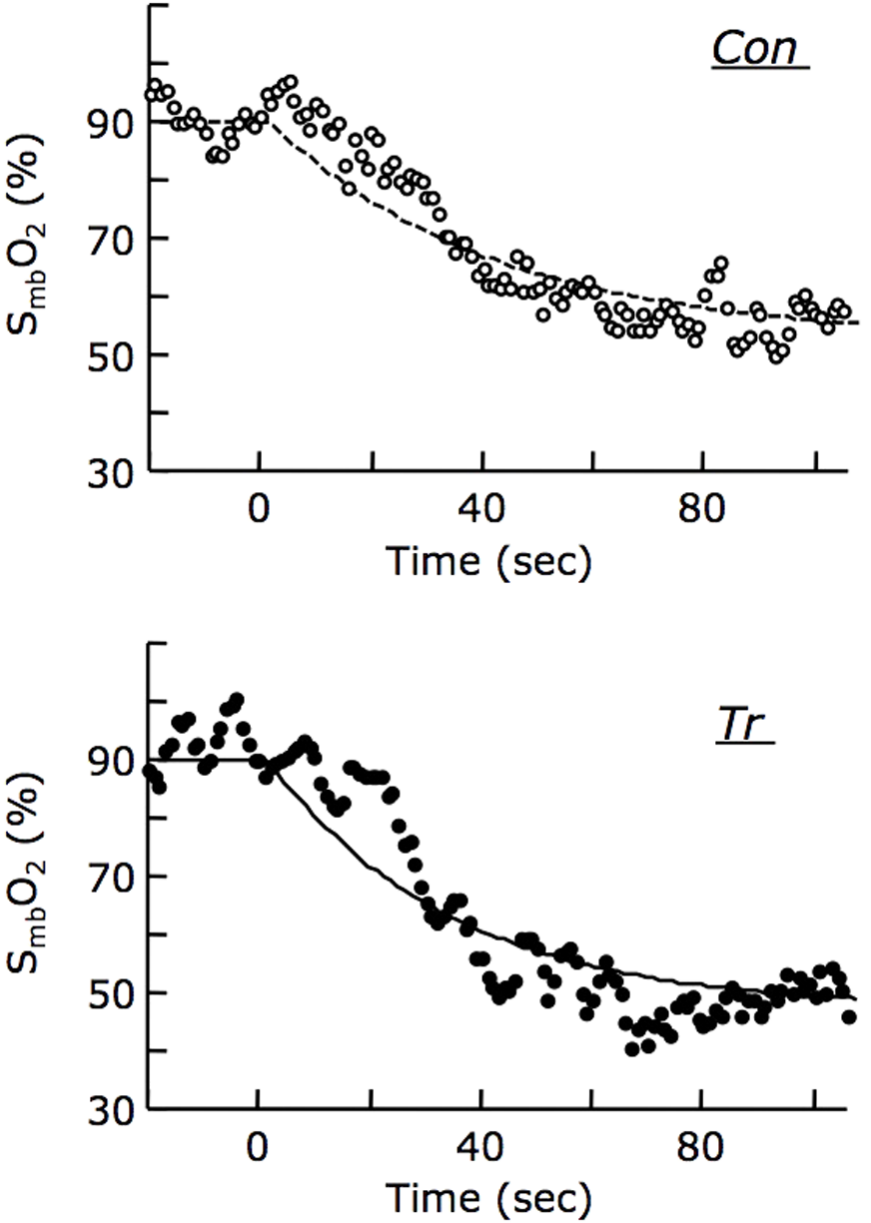
Representative kinetics of myoglobin (Mb) saturation (S_mb_O_2_) during maximal twitch contraction (1 Hz) in the training (Tr) and control (Con) groups. The plots of S_mb_O_2_ show representative data at the maximal twitch tension from the single experiment in each group. While the S_mb_O_2_ kinetics (dotted line) in the representative control rat declined with a mean response time (MRT) of 39.5 sec (upper panel), the S_mb_O_2_ kinetics (solid line) in the representative trained rat declined with an MRT of 33.0 sec (lower panel). The MRT in the S_mb_O_2_ kinetics was shortened by 5 sec on average due to endurance training. By contrast, the S_mb_O_2_ value at steady state did not show any significant difference between the two groups.

Figure 1 shows the representative kinetics of S_mb_O_2_ in each group. As for the S_mb_O_2_ kinetic parameters, the steady-state value, amplitude (AP), and mean rate of change to 63% of the AP value (_0.63_AP/mean response time [MRT]) increased as work intensity increased in both groups, whereas MRT tended to accelerate in both groups. At maximal tension, the steady-state value and AP of S_mb_O_2_ kinetic parameters in the Tr group were unchanged, but _0.63_AP/MRT of the kinetic parameters for S_mb_O_2_ increased. The MRT also tended to be faster in the Tr group. At submaximal tension levels, the steady-state value, AP, and _0.63_AP/MRT in the Tr group were also unchanged, but the MRT of the kinetic parameters for S_mb_O_2_ tended to be faster. The kinetic parameters for P_mb_O_2_ showed that the steady-state value, AP, and _0.63_AP/MRT increased, and the MRT became faster in both groups as work intensity increased. When the kinetic parameters for P_mb_O_2_ were compared at the same relative tension level between both groups, the relative temporal parameters for P_mb_O_2_ kinetics in the trained muscle showed a tendency to accelerate to a higher level. In the present study, while the MRT was used to describe the overall dynamics of S_mb_O_2_ and P_mb_O_2_ fall following the onset of muscle contraction, _0.63_AP/MRT is the effective temporal parameter to show deoxygenation rate of Mb-O_2_ per unit time in the initial phase of muscle contraction. The _0.63_AP/MRT would reflect the steep change in mitochondrial oxygen demand, because we previously reported that _0.63_AP/MRT increased in response to change in mitochondrial oxygen demand due to muscle contraction. Kindig et al.^12^ also used AP/time constant in intracellular PO_2_ kinetics during muscle contraction as an index of initial metabolic response. As for _0.63_AP/MRT parameter in the present study, while its value showed significant difference at 100% of the maximal twitch tension by endurance training, it did not differ at 50% and 75% of the maximal twitch tension between Con and Tr group. This result at submaximal tension level might be caused by non-significant difference in O_2_ demand level during muscle contraction at the relative same intensity between groups. Δm
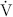
O_2_ did not actually show the significant difference at the 50% and 75% of the maximal twitch tension between groups. On the other hand, at the maximal twitch tension level, _0.63_AP/MRT parameter and Δm
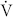
O_2_ in the trained muscle showed significant difference compared with that in the control muscle.

**Figure 2:**
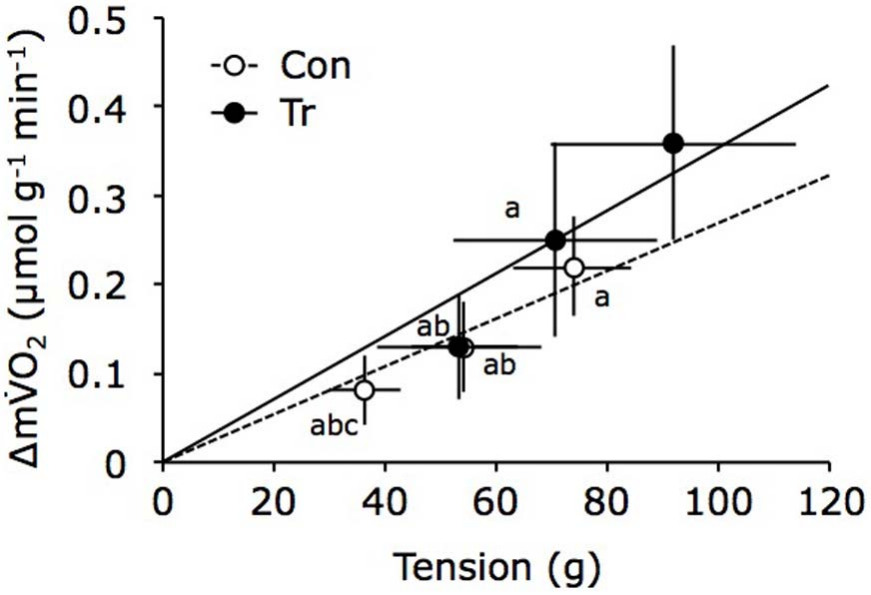
Relationship between muscle tension and Δm
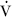
O_2_ during twitch contraction in the training (Tr) and control (Con) groups. Changes in muscle O_2_ uptake (Δm
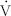
O_2_) due to muscle contraction increased linearly as a function of muscle tension in both groups. Regression lines are based on mean values (n = 9 in each group; Con: Δm
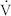
O_2_ = 0.003 × Tension, R^2^ = 0.98, p < 0.05; Tr: Δm
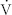
O_2_ = 0.004 × Tension, R^2^ = 0.99, p < 0.05). The data represent the mean ± standard deviation values. The superscript indicates a significant difference (^a^: vs. Tr × 100%, p < 0.05; ^b^: vs. Tr × 75%, p < 0.05; ^c^: vs. Con × 100%, p < 0.05).

Figure 2 shows the relationship between muscle tension and the net increase in m
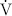
O_2_ during muscle contraction for both groups. While muscle tension and Δm
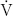
O_2_ were significantly correlated in both groups, the mean individual slope in the Tr group (0.36 ± 0.11 × 10^−2^ μmol/[g^2^·min]) tended to be higher than that in the Con group (0.27 ± 0.06 × 10^−2^ μmol/[g^2^·min]; p = 0.058).

**Figure 3:**
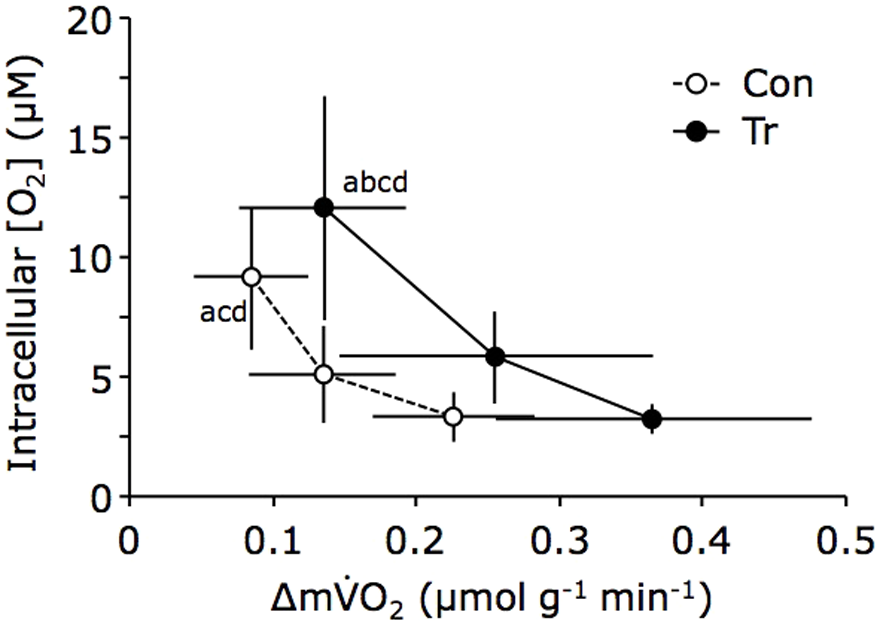
Relationship between intracellular [O_2_] and Δm
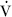
O_2_ during twitch contraction in the training (Tr) and control (Con) groups. Intracellular [O_2_] (in μM) decreased gradually with the increase in changes in muscle O_2_ uptake (Δm
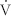
O_2_) in both groups. The relationship between intracellular [O_2_] and Δm
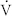
O_2_ was shown as a line graph. Each data point represents the mean ± standard deviation. The superscript letters indicate significant differences (^a^: vs. Tr × 100%, p < 0.05; ^b^: vs. Tr × 75%, p < 0.05; ^c^: vs. Con × 100%, p < 0.05; and ^d^: vs. Con × 75%).

Figure 3 shows the relationship between intracellular [O_2_] and Δm
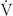
O_2_ during muscle contraction. Intracellular [O_2_] was based on the S_mb_O_2_–P_mb_O_2_ equilibrium. In the present study, the S_mb_O_2_ at rest was assumed to be 90%. Intracellular [O_2_] decreased markedly from 29.2 μM at rest to 9.2 ± 3.0, 5.1 ± 2.1, and 3.3 ± 1.0 μM at 50%, 75%, and 100% of maximal contraction in the Con group, respectively; and from 29.2 μM at rest to 12.1 ± 4.7, 5.8 ± 1.9, and 3.2 ± 0.7 μM at 50%, 75%, and 100% of maximal contraction in the Tr group, respectively. Although intracellular [O_2_] decreased markedly with the Δm
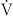
O_2_ in both groups, the Tr group curve showed a smaller [O_2_] decline. At the same level of intracellular [O_2_] in the muscle cell, Δm
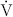
O_2_ in the trained muscle was higher than in the control muscle, suggesting that the trained muscle had more oxidative potential capacity compared with the control muscle.

**Figure 4:**
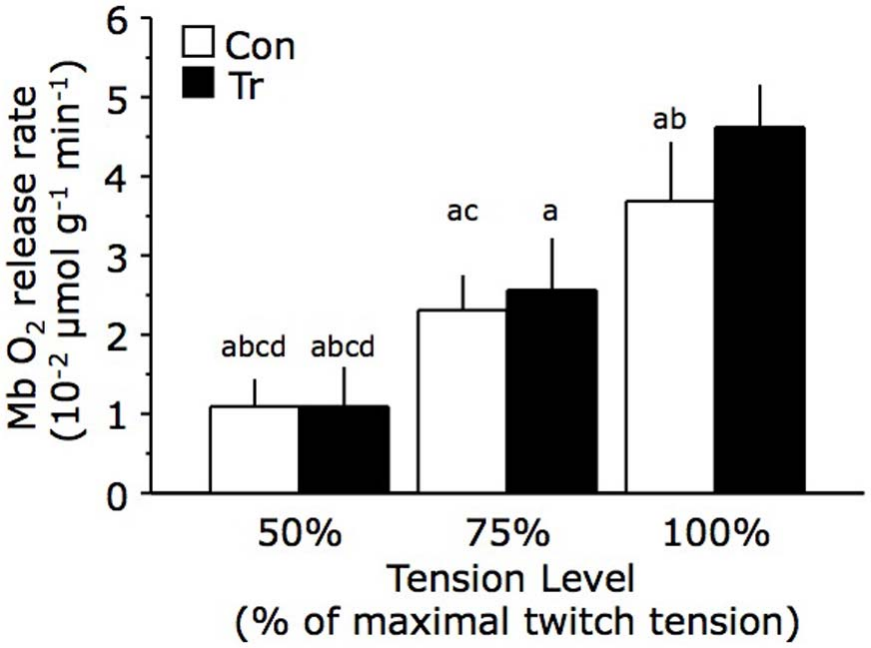
The O_2_ release rate from myoglobin (Mb) at the each tension level in the training (Tr) and control (Con) groups. At the onset of muscle contraction, Mb released its bound O_2_ to the mitochondria with the twitch tension level. The data show the mean ± standard deviation values. The superscript letters indicate significant differences (^a^: vs. Tr × 100%, p < 0.05; ^b^: vs. Tr × 75%, p < 0.05; ^c^: vs. Con × 100%, p < 0.05; and ^d^: vs. Tr × 50%, p < 0.05).

Figure 4 shows the O_2_ release rate from Mb at same percent of MVC in the Tr and Con groups. The O_2_ release rate from Mb increased progressively with the twitch tension level as follows: 1.1 ± 0.3, 2.3 ± 0.4, and 3.7 ± 0.8 × 10^−2^ μmol/(g·min) at 50%, 75%, and 100% of maximal contraction in the Con group, respectively; and 1.1 ± 0.5, 2.6 ± 0.7, and 4.6 ± 0.5 × 10^−2^ μmol/(g·min) at 50%, 75%, and 100% of maximal contraction in the Tr group, respectively. At maximal tension, the O_2_ release rate from Mb showed a significant increase in the Tr group, suggesting more O_2_ supply from Mb to the mitochondria at the onset of muscle contraction.

## Discussion

### Effect of endurance training on muscle oxidative capacity

In the present study, 4 weeks of swimming endurance training resulted in an increase in m
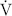
O_2_ peak, even when O_2_ delivery to the endurance-trained hind limb was not greater than that supplied to sedentary muscles. This increase in m
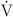
O_2_ peak value at constant flow was consistent with previous studies^2^,^15^. This increase in m
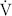
O_2_ peak value without increase in O_2_ delivery to the hind limb muscle would be caused by increased of both O_2_ supply capacity to the mitochondria and O_2_ utilization capacity such as capillary density, mitochondrial respiration capacity, and Mb function in the active muscle. At the equivalent muscle tension, the Tr group showed a slightly higher Δm
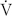
O_2_ than the Con group (Fig. 2). As reflected by a higher CS activity, the endurance-trained muscle had higher muscle oxidative potential. The increase in O_2_ consumption and O_2_ cost at a given work rate would imply a shift to more aerobic metabolism during muscle contraction.

The decrease in L/P at the maximal twitch tension also suggested a greater capacity to oxidize carbohydrate and a tightening in the coupling between ATP supply and demand^13^,^14^. This tight integration of ATP supply and demand is associated with less stimulation of glycolysis, resulting in a decrease in lactate production and a lower cytosolic redox state, and thus an improved coupling between pyruvate oxidation and glycolytic flux^13^,^15^. Collectively, the swimming endurance training in the present study enhanced muscle oxidative capacity, in agreement with evidence previous studies^1^,^16^,^17^.

### Relationship between P_mb_O_2_ kinetics and muscle oxygen consumption

Endurance training increases both m
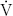
O_2_ peak and Δm
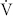
O_2_ at the same percentage of MVC. However, both control and trained muscle show a steady decline in P_mb_O_2_ with increasing MVC. The declining P_mb_O_2_ and the increasing O_2_ consumption indicates an expansion of the O_2_ gradient across the plasma membrane. Both the O_2_ diffusion conductance (DO_2_) and O_2_ gradient between P_cap_O_2_ and P_mb_O_2_ can influence the O_2_ flux into the cell, which then supports the m
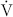
O_2_. In our perfusion model, the O_2_ diffusion conductance would show little change even at the onset of contraction. Fick's first law of diffusion relates explicitly the change in substance concentration over time depends upon the gradient of concentration over space. In a one-dimension case for O_2_ diffusion in the x-direction, the equation clearly states that:
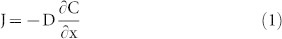


J = diffusion flux (amount of O_2_ crossing a unit area per unit time), D = diffusion coefficient (length of unit area squared x time^−1^), 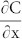 = the change in O_2_ concentration along dimension x or the O_2_ gradient along the x-direction. ^1^H-NMR experiments show Mb desaturating and the cellular PO_2_ decreasing rapidly upon the initiation of muscle contraction^5^,^6^. The debate remains whether with increasing exercise intensity and associated increasing respiration, does the gradient expand or does it reach a plateau. Our experiment data show the gradient expanding. Conductance may still contribute to O_2_ transport into the cell. However, our experiments provide no supporting or contradictory data. They only show that blood flow has not changed. Indeed, we used the constant flow mode and an Hb-free Krebs-Henseleit buffer as perfusate in this perfusion experiment. By using the constant flow mode, the perfusion pressure remained constant throughout the perfusion period, which suggested the convective O_2_ delivery did not change both at rest and during muscle contraction. Additionally, based on the fact that a 30-min equilibrium period elicited a flow-induced vasodilatation corresponding to the given flow rate^18^, we assumed that convective O_2_ delivery was maintained during the perfusion period. Also, as the perfusate did not contain an Hb in this study, muscle contraction would have little affect to skeletal muscle capillary hemodynamic in contrast to the previous study of Kindig et al.^19^ that muscle contraction induced rapid increase of RBC flux and velocity, and capillary hematocrit at the microcirculatory level. Thus, the supposition of a requisite and predominant conductance role in diffusion cannot undermine the observation of an expanding O_2_ gradient that coincides with an increased m
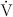
O_2_.

Because the steady-state P_cap_O_2_ level doesn't change^20^ and the P_mb_O_2_ decreases with increasing exercise intensity, the increase in Δm
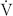
O_2_ and m
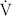
O_2_ peak in the Tr group must also have a contribution from an increased capillarization and/or a greater DO_2_^1^. In the extracellular segment, the angiogenesis of the muscle capillary in the hind limb muscles during training would enhance the supply of O_2_. An increase in capillarity would improve blood flow-to-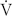
O_2_ relationships within the muscle, allowing for a greater O_2_ extraction^1^. The change in capillarity can also affect DO_2_. A greater DO_2_ after exercise training implies a decreased mean O_2_ diffusion distance from the tissue capillaries to the muscle mitochondria^21^,^22^. In the intracellular segment, O_2_ transport to the mitochondria depends upon Mb-mediated O_2_ flux and free O_2_. The Mb-mediated O_2_ flux in the trained muscle would show a greater value at a relatively higher tension level where intracellular O_2_ tension (equivalent with P_mb_O_2_) became lower, because an adaptive increase in Mb concentration led to the increase in Mb-O_2_ flux, as also shown by a previous study^23^. Therefore, increased augmentation of capillarity and Mb-mediated O_2_ flux during muscle contraction can enhance the capacity supplying O_2_ to the mitochondria to support the increased Δm
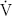
O_2_ and m
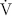
O_2_ peak after endurance training. The experiment results show that the hypothesized expansion of O_2_ gradient due to further decrease in P_mb_O_2_ does not explain the overall increase in Δm
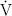
O_2_ and m
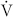
O_2_ peak in endurance-trained muscle.

### O_2_ release rate from Mb at the onset of muscle contraction

Endurance training usually results in faster 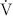
O_2_ kinetics^24^, which will presumably experience result in a smaller decrease in muscle phosphocreatine concentration, a smaller increase in lactate and proton (H^+^) production, and a reduced degradation of muscle glycogen, compared with an individual with slow 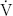
O_2_ on-kinetics^25^,^26^,^27^. Improvement in mitochondrial respiration capacity itself would largely contribute to this adaptation as a result of endurance training. However, no study has investigated the O_2_ supply to the mitochondria at the intracellular level.

We previously found that Mb supplied O_2_ immediately at the onset of muscle contraction and that the O_2_ release rate from Mb increased linearly as the O_2_ demand increased^4^,^28^. These facts suggest that Mb provides an immediate O_2_ source for the sudden increase in m
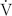
O_2_ at the onset of muscle contraction. The present study reveals an increase in the O_2_ release rate from Mb at the onset of muscle contraction at the maximal twitch tension after endurance training. Myocyte experiments have also suggested that a direct Mb-mediated oxygen delivery might contribute to mitochondrial respiration^29^. The blockade of Mb oxygen-binding capacity suppressed approximately 70% of mitochondrial respiration, even under the condition of sufficiently available O_2_^29^. Indeed, the fact that the O_2_ release rate from Mb at the onset of muscle contraction increases progressively as O_2_ demand increases might indicate that Mb-supplied O_2_ may directly influence m
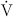
O_2_ kinetics^4^. Taking these findings together, both mitochondrial respiration capacity and O_2_ release rate from Mb might be important factors that regulate m
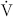
O_2_ kinetics at the onset of muscle contraction.

The binding of O_2_ to Mb and Hb certainly proceeds much faster than transport^30^,^31^, even though the rate-determining step depends on a much slower off rate. But, dismissing any contribution of Mb in regulating respiration in the cell (an inhomogeneous and compartmentalized system) based on just the steady-state rate determining step argument seems tenuous. If the blood delivers a sufficient O_2_ supply, the cell would not need to withdraw O_2_ from its Mb reservoir at the start of muscle contraction. But the cell does withdraw O_2_ from Mb, as our data and all ^1^H-NMR data show, and takes a finite amount of time to reach a new steady state^5^,^6^. Thermodynamics requires a demand to elicit the loss of O_2_ from Mb, and both ATP utilization and respiration surge once contraction starts. The kinetics coincides with O_2_ release from Mb. Thus, the postulated regulatory relationship between the O_2_ release rate from Mb and m
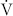
O_2_ seems quite reasonable and consistent with the postulated role of Mb. Once Mb desaturation has reached a new steady state, vascular O_2_ supply must begin to contribute significantly to sustain the rising m
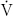
O_2_. To avoid the missteps in the rate limiting step approach, metabolic control theory vantage advocates examining the relative contribution of MbO_2_ and O_2_ to the regulation of m
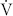
O_2_. Note that Mb never resaturates to its control level as long as the muscle contraction is sustained. The cellular PO_2_ drops during contraction, consistent with an enhanced O_2_ gradient.

The O_2_ release rate from Mb reflects the intracellular m
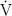
O_2_^5^,^6^. Consequently, the enhanced intracellular m
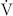
O_2_ observed after endurance training could be induced by a 30% increase in [Mb] concentration and a 12% acceleration in the Mb deoxygenation rate at the maximal twitch tension. Tables 2 and 3 show that an increase in [Mb] predominantly contributes to an increase in O_2_ release rate from Mb in the trained skeletal muscles. Although several studies have reported that endurance training produces an increased [Mb] in rat limb muscle^16^,^17^,^32^,^33^, the physiological significance of increased [Mb] has not been demonstrated *in vivo*. The present study also demonstrated that the deoxygenation rate of Mb became faster at the onset of muscle contraction after endurance training, suggesting a more efficient O_2_ transport from Mb to the mitochondria at the transient phase.

In addition, the P_mb_O_2_ response at the onset of muscle contraction showed the tendency to be faster after endurance training in the present study. Hirai et al.^20^ reported that endurance training led to slower P_cap_O_2_ kinetics during 1-Hz twitch contraction, indicating a relatively greater increase in muscle blood flow at the microvascular level than in O_2_ diffusivity. This adaptation would be mainly caused by an increase in capillary density. Meanwhile, the intracellular O_2_ environment was reported to adjust more effectively to the abrupt increase in oxygen demand at the onset of muscle contraction, before the microcirculatory O_2_ environment has adapted^4^. In fact, the MRT of P_mb_O_2_ kinetics became approximately 5 seconds faster on average by endurance training. The acceleration of P_mb_O_2_ kinetics with slower P_cap_O_2_ kinetics would imply a sharper expansion of O_2_ gradient at the onset of muscle contraction, resulting in a more efficient O_2_ transport from Mb to the mitochondria at the transient phase.

In the present study, we have performed additional statistical analyses on kinetics parameters to check the existence of type II error. As for MRT in P_mb_O_2_ kinetics, the effect size was 0.052, and the statistical power was 0.109. This level of statistical power implies the existence of a type II error. However, based on our experimental content, increasing the sample size would not necessarily improve the accuracy or precision of our results. This might be one of limitations in this type of experiment. Actually, although significant difference was not recognized for kinetics parameters such as MRT between groups at the relative same tension level, the shorting of MRT by 5 sec on average at the maximal tension level in both of S_mb_O_2_ and P_mb_O_2_ kinetics suggests the possibility that swimming endurance training accelerates both kinetics during muscle contraction.

In summary, the results presented herein suggest that Mb plays an important role in the faster m
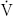
O_2_ response and increased m
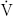
O_2_ in trained skeletal muscles. However, how Mb-bound O_2_ is supplied to the mitochondria at the onset of muscle contraction remains unclear. Recently, Yamada et al.^34^ suggested the possibility that the presence of Mb in mitochondrial fractions indicates involvement in the immediate O_2_ release from Mb at the onset of muscle contraction. If so, endurance training might impact Mb localization in the muscle cell. Further research is required to elucidate the mechanism of O_2_ transport to the mitochondria within muscle cells and the causal relationship between cellular factors altering the O_2_ off rate in Mb and mitochondrial respiration activity.

## Methods

### Experimental Animals and Preparation of Hindlimb Perfusion

Male Wistar rats were employed as subjects. All were housed in a temperature-controlled room at 23 ± 2°C with a 12-h light–dark cycle and maintained on a commercial diet with water ad libitum. The procedures conformed to the “Fundamental Guidelines for Proper Conduct of Animal Experiment and Related Activities in Academic Research Institutions” (published by the Ministry of Education, Culture, Sports, Science and Technology, Japan) and was approved by the Ethics Committee for Animal Experimentation of Kanazawa University (Protocol AP-101636).

Five-week-old Wistar rats were randomly divided into the Con and 4 weeks Tr groups (n = 9 in each group). The training protocol for the swimming group was as follows: On the first and second days, the rats swam for 1 h in two 30-min bouts separated by 5 min of rest. On the third and fourth days, the rats swam for 1.5 hours in three 30-min bouts separated by 5 min of rest. On and after the fifth day, the rats swam for 2 hours in four 30-min bouts separated by 5 min of rest. Except for the first bout of swimming training until the sixth day, a weight equal to 2% of the rats' body weight was tied to the bodies of the rats. The rats performed the above swimming protocol six days per week. During swimming exercise, the water temperature was kept at around 35°C. The tank's shape was square and its characteristics were 48 cm depth, 80 cm longitudinal and 60 cm width. All rats swam in that tank and an average surface area of at least 600 cm^2^/rat. Also, we kept monitoring to prevent the climbing, diving and bobbing of rat during swimming training. In cases where these behaviors were observed, they were dealt with immediately.

After 4 weeks (at 9-week of age), hindlimb perfusion was performed in each group (Con group: initial body weight (BW) at 5 weeks old; 143–168 g, final BW at 9 weeks old; 257–295 g, Tr group: initial BW; 140–176 g, final BW; 226–250 g). Preparation of isolated rat hindlimb and the perfusion apparatus are described in previous reports^4^,^28^. All surgical procedures were performed under pentobarbital sodium anesthesia (45 mg kg^−1^ intraperitoneal). The rats were killed by injecting 1 M KCl solution directly into the heart, followed by a surgical procedure, and an Hb-free Krebs-Henseleit buffer (NaCl, 118 mM; KCl, 5.9 mM; KH_2_PO_4_, 1.2 mM; MgSO_4_, 1.2 mM; CaCl_2_, 1.8 mM; NaHCO_3_, 20 mM; Glucose, 15 mM) equilibrated with 95%O_2_ + 5%CO_2_ at 37°C was perfused into the abdominal aorta in flow through mode, at a constant flow rate. In order to adjust the perfusion pressure to approximately 80.0 mmHg, the flow rate was set to 22.0 ± 0.0 ml min^−1^ in the Con group and 22.1 ± 0.9 ml min^−1^ in the Tr group. In this condition, the average perfusion pressures were 78.6 ± 7.7 mmHg in the Con group and 74.2 ± 5.2 mmHg in the Tr group. Therefore, the perfusion resistance was unchanged throughout the perfusion period. In addition, no sign of oedema in the hindlimb was seen at the given flow rate. The effluent was collected from the inferior vena cava in order to measure m
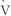
O_2_ and the lactate and pyruvate concentrations.

### Measurement Parameters

The twitch contraction protocol and measurement of Mb oxygenation and m
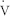
O_2_ followed the previous methods^4^,^28^. The sciatic nerve of the left hindlimb was then exposed and connected to two parallel stainless steel wire electrodes (Unique Medical, Tokyo, Japan) and the Achilles' tendon was connected to a sensitive strain gauge with a string (MLT500/D, AD Instrument, Castle Hill, NSW, Australia). The stimulation pulse via the sciatic nerve derived by an electrostimulator system (Model RU-72, Nihon Koden, Tokyo, Japan) was 1 Hz in frequency (delay, 10 μsec; duration, 1 msec) for 120 sec (120 twitch contractions). Target tension was controlled by changing the voltage of stimuli to obtain 50%, 75% and 100% of peak tension under buffer-perfused conditions (3–8 volts). Twitch tension was calculated as the average of a series of contractions. The muscle also showed no sign of fatigue, even at the highest stimulation intensity.

An NIRS instrument (NIRO-300 + Detection Fibre Adapter Kit, Hamamatsu Photonics, Shizuoka, Japan) was employed to measure oxygenation of Mb at rest and during muscle contraction. The distance between the photodiode and the LED was fixed at 10 mm. The toe of the foot was secured by a clamp with the rat laid on its back. After that, the NIRS probes were firmly attached to the skin of the gastrocnemius muscle and were fixed by clamps on both sides of the muscle. During the initial period, for at least 30 sec before the start of contraction, the average fluctuation in the NIRS signals was adjusted to a reference value of zero. After the exercise protocol, the anoxic buffer (equilibrated with 95% N_2_ + 5% CO_2_ gas) was perfused for 30 min to obtain maximal Mb desaturation. The muscle then received electrical stimulation to contract for 2 min. No further increase in change in NIRS signal associated with concentration of deoxygenated Mb (Δ[deoxy-Mb]) signal was evident. The final Δ[deoxy-Mb] signal intensity served as the normalization constant for 100% Mb deoxygenation.

m
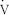
O_2_ (μmol g^−1^ min^−1^) was calculated from the arteriovenous O_2_ content differential multiplied by flow rate, using two O_2_ electrodes (5300A, YSI, Yellow Springs, Ohio, USA). Two O_2_ electrodes were adjusted for the vapour pressure of water before hindlimb perfusion experiment. The oxygen cost was calculated by dividing change in m
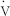
O_2_ due to muscle contraction by muscle tension. The L/P remains constant and shows no significant increase from the resting muscle value. Lactate levels can increase for a number of reasons, including lack of adequate O_2_ delivery. Therefore, limited oxygen availability should lead to an increase in lactate level as anaerobic glycolysis starts. Indeed, the L/P increases during perfusion with anoxia buffer (95%N_2_) increases. O_2_ availability and delivery can sustain maximal contraction with no sign of fatigue.

The sampling rate for the NIRS data was 1 Hz. The other parameters (tension, perfusion pressure, O_2_ content at the inflow and outflow) were collected using a data acquisition system (PowerLab 8SP, AD Instruments, Australia) at a sampling rate of 1 kHz. All the data were transferred to a personal computer with acquisition software (Chart ver. 5.5.6. AD Instruments).

### Data Analysis

The data analysis followed our previous methods^4^. A simple moving average smoothed the Δ[deoxy-Mb] and Δ[oxy-Mb] NIRS signals using a rolling average of 5 points, which corresponds to a 5-sec timeframe^35^. The Δ[deoxy-Mb] signals were calibrated against two different NIRS signal values: one at rest as 10% Mb deoxygenation and the other during steady state with anoxic buffer perfusion as 100% Mb deoxygenation. While the S_mb_O_2_ at rest could not be determined by NIRS, the value was assumed to be 90% based on previous studies reporting that the S_mb_O_2_ at rest was greater than 90%^5^,^36^.

The %Δ[deoxy-Mb] plots were converted to S_mb_O_2_ (%) plots using the following equation:
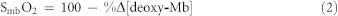


S_mb_O_2_ plots were fitted by the following single-exponential equation to calculate kinetics parameters using an iterative least-squares technique by means of a commercial graphing/analysis package (KaleidaGraph 3.6.1, Synergy Software, Reading, PA, USA):
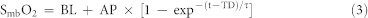
where BL is the baseline value, AP the amplitude between BL and the steady-state value during the exponential component, TD the time delay between onset of contraction and appearance of S_mb_O_2_ signals, and τ the time constant of S_mb_O_2_ signal kinetics. MRT calculated by TD + τ was used as an effective parameter of the response time for Mb deoxygenation at onset of muscle contraction. Dividing 63% of AP by MRT yields a value for the time-dependent change in Mb deoxygenation. These parameter, _0.63_AP/MRT, for S_mb_O_2_ shows the O_2_ release rate from Mb, which indicates the amount of O_2_ released from Mb per unit time at onset of exercise. The O_2_ release rate from Mb was calculated using the following equation:
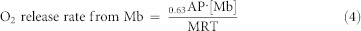
where _0.63_AP/MRT for S_mb_O_2_ was the Mb deoxygenation rate in %/sec. Inserting this value for Mb into the equation led to determination of the O_2_ release rate from Mb in micromoles per gram per minute.

We reconstructed P_mb_O_2_ kinetics based on the resulting S_mb_O_2_ kinetics parameters. The model S_mb_O_2_ kinetics was converted to P_mb_O_2_ (mmHg) using the following equation:
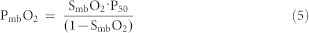
where P_50_ is the partial oxygen pressure required to half-saturate Mb. A P_50_ of 2.4 mmHg was used for this equation, assuming a muscle temperature of 37°C^37^. The calculated P_mb_O_2_ plots were evaluated to obtain an MRT of its kinetics using the same single exponential equation as for P_mb_O_2_. The _0.63_AP/MRT for P_mb_O_2_ indicates a rate of decrease in P_mb_O_2_ at muscle contraction onset. P_mb_O_2_ at steady state was calculated by using the S_mb_O_2_ value at steady state. Since O_2_ partial pressure corresponds to a specific amount of dissolved O_2_, intracellular [O_2_] (μM) was calculated from the P_mb_O_2_ value at rest and at each exercise intensity using the following equation:
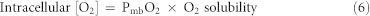
with P_mb_O_2_ is in mmHg, and O_2 _solubility in buffer is 0.00135 μmol ml^−1^ mmHg^−1^ at 37°C^38^.

### Mb Concentration and CS Activity in Buffer-Perfused Muscle Tissue

After buffer perfusion experiment, Mb concentration in muscle tissue was measured by a modified Reynafarje method^39^. CS activity, a mitochondrial enzyme and marker of muscle oxidative potential, was measured in whole muscle homogenates by using the spectrophotometric method of Srere^40^.

### Statistical Analyses

All data are expressed as mean ± SD. Statistical differences were examined using two-way unpaired measures analysis of variance (ANOVA) (tension level × training). A Turkey-Kramer post-hoc test was applied if the ANOVA indicated a significant difference. An unpaired *t*-test was used in comparing biochemical and physiological parameters between groups. Pearson's correlation coefficient was calculated when the relationship between two variables was evaluated. The level of significance was set at *p* < 0.05.

## Author Contributions

H.T., K.M. and T.J. designed the research, H.T., Y.F., T.Y. and M.O. conducted the experiment and analysed the data, H.T. wrote the manuscript, K.M. and T.J. edited paper and T.H., S.I., T.H. and T.I. helped experiments.

## Figures and Tables

**Table 1:** Descriptive data for the muscle weight

Parameter		Unit	Con	Tr
Body Mass		g	269.0 ± 11.6	255.4 ± 22.7
Muscle Mass	m. Gastrocnemius	mg	1711.1 ± 73.8	1541.0 ± 136.7
	m. Plantaris	mg	346.9 ± 15.0	289.3 ± 25.7 *
	m. Soleus	mg	126.4 ± 5.5	98.2 ± 8.7 *
	GPS	mg	2141.6 ± 92.4	1928.4 ± 171.0 *

Values are mean ± SD (n = 9 in each group). Con: control group. Tr: training group. GPS: gastrocnemius-plantaris-soleus. A superscript (*) indicates a significant difference (p < 0.05 vs. Con).

**Table 2:** Contractile and metabolic properties of hindlimb muscles

Parameter	Unit	Con	Tr
Maximal Tension	g	73.7 ± 10.6	92.0 ± 21.9 *
m 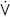 O_2_ at rest	μmol g^−1^ min^−1^	0.48 ± 0.09	0.58 ± 0.13
m 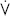 O_2_peak	μmol g^−1^ min^−1^	0.70 ± 0.10	0.93 ± 0.16 *
[Mb]	μmol g^−1^	0.10 ± 0.01	0.12 ± 0.01 *
CS activity	μmol g^−1^ min^−1^	28.4 ± 1.1	40.8 ± 6.7 *
L/P ratio		19.1 ± 2.22	12.5 ± 5.2 *

Values are mean ± SD (n = 9 in each group for physiological parameters, n = 6 in each group for biochemical parameters). Con: control group. Tr: training group. [Mb]: Mb concentration. CS activity: citrate synthase activity. L/P ratio: Lactate to pyruvate ratio measured in effluent perfusate at the maximal twitch tension. As for [Mb] and CS activity, the deep portion of gastrocnemius muscle was used for the measurement as a representative muscle. A superscript (*) indicates a significant difference (p < 0.05 vs. Con).

**Table 3:** Muscle tension, muscle oxygen consumption, S_mb_O_2_ and P_mb_O_2_ kinetics parameters during muscle contraction at each tension level

			Tension Level (% of maximal twitch tension)		
Parameter	Unit	Group	50%	75%	100%
Muscle Tension	g	Con	36.4 ± 6.4^ abc^	54.2 ± 9.6 ^a^	73.7 ± 10.6
		Tr	53.5 ± 15.0 ^a^	70.9 ± 18.3 ^a^	92.0 ± 21.9
Δm 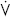 O_2_	μmol g^−1^ min^−1^	Con	0.08 ± 0.04 ^abc^	0.13 ± 0.05 ^ab^	0.22 ± 0.06 ^a^
		Tr	0.13 ± 0.06 ^ab^	0.25 ± 0.11 ^a^	0.36 ± 0.11
O_2_cost	10^−2^ μmol g^−2^ min^−1^	Con	0.22 ± 0.08^ a^	0.25 ± 0.10 ^a^	0.30 ± 0.08
		Tr	0.25 ± 0.08 ^a^	0.35 ± 0.13	0.40 ± 0.12
S_mb_O_2_ kinetics					
Steady-State Value	%	Con	72.7 ± 6.5 ^acd^	58.7 ± 10.9 ^c^	49.0 ± 8.1 ^b^
		Tr	76.8 ± 7.9 ^abd^	62.7 ± 7.9 ^a^	49.5 ± 5.3
AP	%	Con	−19.2 ± 7.0 ^acd^	−31.2 ± 11.0 ^c^	−41.0 ± 8.1 ^b^
		Tr	−13.2 ± 7.9 ^abd^	−27.3 ± 7.9^ a^	−40.5 ± 5.3
MRT	s	Con	63.0 ± 18.2 ^ac^	52.2 ± 14.0	43.7 ± 6.6
		Tr	52.6 ± 12.4	48.3 ± 10.2	39.3 ± 5.9
_0.63_AP/MRT	% s^−1^	Con	−0.18 ± 0.06 ^abcd^	−0.37 ± 0.07 ^ac^	−0.58 ± 0.10 ^a^
		Tr	−0.15 ± 0.07 ^abcd^	−0.36 ± 0.10 ^a^	−0.65 ± 0.08
P_mb_O_2_ kinetics					
Steady-State Value	mmHg	Con	6.9 ± 2.2 ^ac^	3.8 ± 1.5	2.4 ± 0.8
		Tr	8.9 ± 3.4 ^abcd^	4.3 ± 1.4	2.4 ± 0.5
AP	mmHg	Con	−15.0 ± 2.3 ^ac^	−17.6 ± 1.8	−19.1 ± 0.7
		Tr	−12.6 ± 3.4 ^abcd^	−17.3 ± 1.4	−19.2 ± 0.5
MRT	s	Con	43.8 ± 10.3 ^abc^	34.6 ± 5.6	29.5 ± 5.7
		Tr	43.2 ± 9.4 ^ac^	33.7 ± 4.6	25.9 ± 5.2
_0.63_AP/MRT	mmHg s^−1^	Con	−0.23 ± 0.06 ^abcd^	−0.33 ± 0.06 ^a^	−0.42 ± 0.07
		Tr	−0.19 ± 0.06 ^abcd^	−0.23 ± 0.06 ^ac^	−0.47 ± 0.09

Values are mean ± SD (n = 9 in each group). Con: control group. Tr: training group. Δm
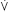
O_2_ is the net increase in m
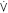
O_2_. AP is the amplitude between BL (baseline) and the steady-state value during the exponential component. MRT is the time required to reach 63% of AP from the onset of muscle contraction. _0.63_AP/MRT is calculated by dividing _0.63_AP by MRT. Superscripts indicate a significant difference (a: p < 0.05 vs. Tr × 100%; b: p < 0.05 vs. Tr × 75%; c: p < 0.05 vs. Con × 100%; d: p < 0.05 vs. Con × 75%).
